# Adherence to hypertension and dyslipidemia treatment and its implication on control of cardiovascular disease in Vietnam: A semi-systematic review

**DOI:** 10.1097/MD.0000000000032137

**Published:** 2022-12-23

**Authors:** Pham Manh Hung, Vu Huy Thanh, Hoang Van Sy, Dang Quy Duc, Vuong Anh Tuan, Anh T Q Tran, Grace E Brizuela, Hieu B Tran

**Affiliations:** a Vietnam Heart Institute, Bach Mai Hospital, 78 Giai Phong, and Hanoi Medical University, Hanoi, Vietnam; b Vietnam Heart Institutes, Bach Mai Hospital, Hanoi, Vietnam; c Cardiovascular Center, Cho Ray Hospital, 201B Nguyen Chi Thanh, and Internal Faculty, University of Medicine and Pharmacy at Ho Chi Minh City, Ho Chi Minh City, Vietnam; d Cardiovascular Center, Cho Ray Hospital, Ho Chi Minh City, Vietnam; e Viatris Vietnam Limited, Ho Chi Minh City, Vietnam; f Research, Development and Medical, Viatris, Makati, Metropolitan Manila, Viatris, Manila, Philippines; g Coronary Care Unit, Vietnam Heart Institutes, Bach Mai Hospital, 78 Giai Phong, Hanoi, Vietnam.

**Keywords:** adherence, cardiovascular disease, dyslipidemia, hypertension

## Abstract

To understand the prevalent issues and challenges in the provision of care for dyslipidemia and hypertension in Vietnamese adults, quantification of patient journey stages (awareness, screening, diagnosis, treatment, adherence, and control) was performed in this semi-systematic review.

**Methods:**

The EMBASE and MEDLINE databases were searched for English articles published between 2010 and 2019. Thesis abstracts, letters to the editor, editorials, case studies, and studies on patient subgroups or nationally unrepresentative studies, were excluded. Articles from Google, the Incidence and Prevalence Database, the World Health Organization, Vietnam’s Ministry of Health, and those suggested by the authors were also included. The last search was run on December 10, 2019 for dyslipidemia and hypertension.

**Results:**

A reviewer independently screened 586 retrievals for dyslipidemia and 177 retrievals for hypertension, and extracted data from 2 articles on dyslipidemia and 6 articles on hypertension that were included in the final synthesis.

**Conclusion:**

The data generated in this review will help overcome these issues and barriers to patient care in populations with these 2 conditions.

## 1. Introduction

Cardiovascular disease (CVD) is the leading cause of morbidity and mortality worldwide ^[1]^. Hypertension and dyslipidemia are the major risk factors for CVD. Both the risk factors have detrimental effects on the vascular endothelium, which results in enhanced atherosclerosis leading to CVD ^[2]^. Globally, an estimated 1.13 billion people have hypertension, with the majority of them living in low-and middle-income countries ^[3]^. Raised cholesterol is estimated to cause 2.6 million deaths and 29.7 million disability-adjusted life years ^[4]^. In Vietnam, CVDs are responsible for 31% of all deaths ^[5]^, while hypertension is the 3^rd^ leading cause of death in hospitalized adults ^[6]^.

Vietnam is witnessing an upsurge in the prevalence of hypertension and dyslipidemia due to economic development, aging population, urbanization, and changes in dietary habits and lifestyle ^[7]^. To address this, Vietnam’s healthcare system provides services at the community level through commune health stations and village health workers. These grassroots-level providers help in implementing National Target Programmes such as Vietnam’s National Hypertension Programme. They conduct population-wide screening campaigns for early detection of hypertension in people aged > 40 years, link the needy to primary care physicians (PCPs) or specialists, and spread awareness regarding hypertension among common people ^[8]^.

The unique milieu of various countries highlights that there is a need to identify locally relevant issues in care provision to formulate locally effective solutions for coping with increasing demands on the healthcare system due to rising non-communicable diseases. Also, preventive efforts have limited effectiveness unless they are based on local guidance ^[8]^. The current status of care provision in Vietnam and issues contributing to it remain unclear, despite all the efforts being made. To understand how the issues and barriers at grassroots level and local challenges in the healthcare system contribute to the current status of care provision for hypertension and dyslipidemia in Vietnam, a methodology of quantifying patients completing stages of their journey (awareness, screening, diagnosis, treatment, adherence, and control) of interactions with the healthcare system was deployed. This methodology is known as mapping the patient journey towards actionable beyond the pill solutions ^[9]^.

The objective of the current semi-systematic literature review was to review publications related to dyslipidemia and hypertension for quantifying patients in different stages of their journey for these 2 conditions, along with health literacy and prevalence; and thereby assess the implication of poor outcomes on the cardiovascular health of the patients.

## 2. Methods

### 2.1. Overview

Adopting the mapping the patient journey towards actionable beyond the pill solutions methodology ^[9]^, studies conducted in Vietnam describing patient journeys with respect to hypertension and dyslipidemia were captured. Quantification of patients at various stages of the journey for dyslipidemia and hypertension was done to reflect upon the overall care for these conditions. It was used as a proxy indicator of availability or accessibility of care that may be affecting the control of both dyslipidemia and hypertension. Methods of conducting the review and eligibility criteria were documented in advance. The definitions used in the review are provided in Table S1, Supplemental Digital Content, http://links.lww.com/MD/I52.

### 2.2. Search strategy

An electronic search was conducted in 2 parts: Structured search using medical subject headings terms on EMBASE and MEDLINE through OVID access; Unstructured search on Google and articles from incidence and prevalence database, world health organization and ministry of health (MoH) of Vietnam.

Keywords used for dyslipidemia were:

(dyslipidemia OR hypercholesterolemia OR cholesterol OR triglycerides OR LDL) AND

(epidemiology OR prevalence OR incidence OR national OR survey OR registry OR Statistics) AND (health literacy OR screening OR awareness OR knowledge OR treated OR treatment OR diagnosis OR undiagnosed OR diagnosed OR therapy OR controlled OR control OR uncontrolled OR adherence OR adhere OR compliance) AND (Malaysia OR Philippines OR Vietnam OR Thailand)

Keywords used for hypertension were:

(hypertension OR blood pressure OR hypertensives) AND (epidemiology OR prevalence OR incidence OR national OR survey OR registry) AND (awareness OR knowledge OR health literacy OR screening diagnosis OR diagnosed OR undiagnosed OR treatment OR treated OR untreated OR control OR controlled OR uncontrolled OR adherence OR compliance OR adhere OR therapy OR non-adherence) AND (Vietnam OR Viet Nam)

The retrieved records were filtered for region, language, availability of full-text (except conference abstract), and human studies. Articles focusing on Vietnam were included and other countries in the region were filtered. Within the structured search results, studies published in English from 2010 to 2019, for dyslipidemia and hypertension were selected. In the unstructured search, articles were selected irrespective of the year of publication. Articles present in duplicate were excluded. The last search for dyslipidemia and hypertension was run on December 10, 2019.

### 2.3. Inclusion and exclusion criteria

Studies were eligible for inclusion if they were: systematic review and/or meta-analysis, randomized controlled study, observational study, narrative reviews (full-texts published and conference abstracts) focusing on dyslipidemia and hypertension; related to adults aged ≥ 18 years old; not restricted to a specific patient subgroup, such as patients with comorbidities or pregnant women; having national representativeness; and reporting quantitative data from the patient journey touchpoints for dyslipidemia and hypertension. Case studies, letters to the editor, editorials, and thesis abstracts were excluded.

### 2.4. Study selection

An independent reviewer conducted a semi-systematic literature search to extract data from both structured and unstructured searches and screened the titles, abstracts, and full-text papers of the retrieved publications against the inclusion and exclusion criteria. A second independent reviewer reviewed the screened studies. Any disagreements were reconciled by discussion among the reviewer and the coauthors. Data gaps were identified and supplemented with studies suggested by authors who are subject matter experts from Vietnam.

### 2.5. Data extraction

Relevant data from the included studies were extracted in an excel sheet, which was validated by local experts, who are also the authors of this paper, to ensure consistency with local prevalent conditions and expert opinion. Data related to various variables were extracted. For a study reporting a low rate for a particular patient journey stage, the factors possibly contributing to the low rate(s), factors associated with the stages of the patient journey, influencers of control of CVDs, and suggested interventions were also extracted from the studies.

### 2.6. Data analysis

Data from the included studies concerning prevalence and patient journey touchpoints, namely, awareness, screening, diagnosis, treatment, adherence, and control of dyslipidemia and hypertension were pooled. Weighted means were estimated for prevalence, diagnosis, treatment, and control of hypertension as there were multiple values represented in the included studies. Computed rates for the patient journey stages are presented as a tabular summary.

### 2.7. Ethical consent

This review is based on previously conducted studies. Hence approval from the Ethics committee is not required.

## 3. Results

### 3.1. Flow of retrieved studies through the review

**Figure 1.**
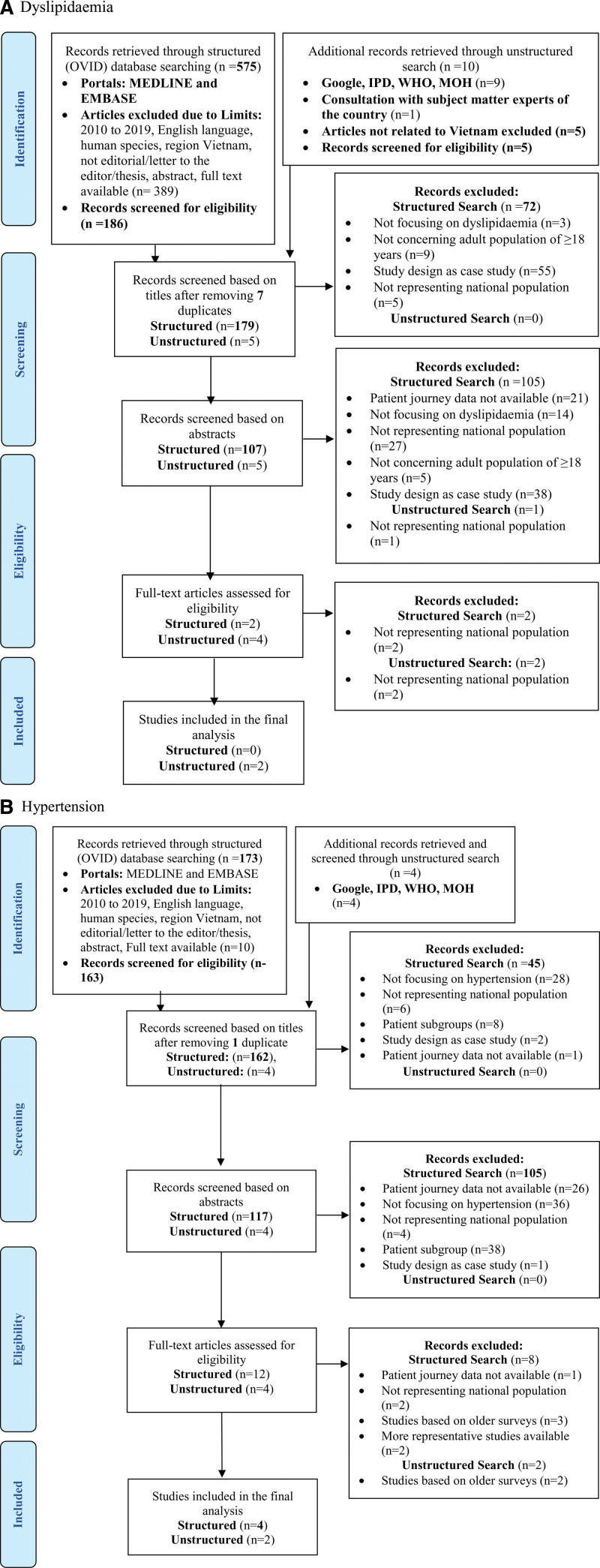
PRISMA diagram showing selection of studies for inclusion in the review. PRISMA = preferred reporting items for systematic reviews and meta-analyses.

Dyslipidemia studies that were retrieved from the structured search were published between 2011 and 2015 while hypertension articles were published between 2015 and 2019. For dyslipidemia, 575 and 10 articles were retrieved from structured and unstructured searches, respectively. Of the 575 articles, 389 were filtered out, as these did not report data from Vietnam. The remaining 186 articles were screened for eligibility for inclusion. Finally, no article from the structured search met the inclusion criteria. Two articles from the unstructured search were included in the final data synthesis ^[10,11]^. For hypertension, 173 articles from the structured search and 4 articles from the unstructured search were retrieved. Of the 173 articles, 5 articles did not involve the Vietnamese population, 4 articles with full text not available, and 1 editorial was filtered out. The remaining 163 articles were screened for eligibility for inclusion. Finally, 4 articles from the structured search and 2 articles from the unstructured search were included in the final data synthesis ^[11–16]^. The flow of the studies through the review was plotted as preferred reporting items for systematic reviews and meta-analyses flow chart (Figs. 1A, 1B). Details of the included studies are provided in Table 1.

**Table 1:** Characteristics of the included studies.

**S. No**	**Authors**	**Year**	**Population**	**Sample size**	**Patient Journey Stages**
**Dyslipidemia**					
1	National Survey on Risk Factors of Non communicable diseases (STEPS) Vietnam 2015, WHO ^[11]^	2015	18–69 yrs	3080	Prevalence (30.2%), screening (25.9%), diagnosis (5.8%), treatment (22.1%)
2	Park *et al* ^[10]^	2011	“Hypercholesterolaemic patients aged > 18 yrs who had		Control (40.1%)
**Hypertension**					
1	WHO NCD Country Profile, Vietnam, 2018 ^[16]^	2018	Vietnamese adults > 18 yrs	22	Prevalence (22%)
2	Cuong *et al* ^[14]^	2019	Vietnamese adults > 18 yrs	2203	Prevalence (24.3%)
3	Meiqari *et al* ^[12]^	2019	Vietnamese adults > 18 yrs	44941	Prevalence (21.1%), diagnosis (9.3%), treatment (4.7%)
4	National Survey on Risk Factors of Non communicable diseases (STEPS) Vietnam 2015, WHO ^[11]^	2015	Vietnamese adults aged 18–69 yr	3856	Prevalence (18.9%), screening (70.5%), diagnosis (11.6%), treatment (24.9%), control (9.7%)
5	Minh *et al* ^[13]^	2019	Vietnamese adults > 18 yrs	10993	Prevalence (28.7%)
6	Poulter *et al* ^[17]^	2019	Adults > 18 yrs	10993	Prevalence (28.7%), treatment (52.1%), control (32.5%)

NCD = non-communicable diseases, STEPS = stepwise approach for NCD risk factor surveillance, WHO = world health organization.

### 3.2. Pooled estimates

Pooled estimates for dyslipidemia and hypertension prevalence were 30.2% and 22% respectively. Overall, quantification of patients in all stages of dyslipidemia and hypertension was low, except for hypertension screening (70.5%) (Table 2). None of the studies provided awareness rates for either of the conditions, though national surveys reported very low diagnosis rates. Similarly, there was no published data available for treatment adherence for either of the conditions.

**Table 2:** Pooled estimates of patient journey touchpoints from the included studies.

**Condition**	**Awareness**	**Screening**	**Diagnosis**	**Treatment**	**Adherence**	**Control**
Dyslipidemia	No data	25.9%	5.8%	22.1%	No data	40.1%
Hypertension	No data	70.5%	9.5%†	14.6%†	No data	31%†

† = Weighted average.

## 4. Discussion

The study aimed to quantify patients with dyslipidemia and hypertension in different stages of their journey, and assess their prevalence, and factors affecting poor prognosis. The data suggested a compromised patient journey for both conditions and that the published data was scarce to draw any valid conclusions. The screening rate for hypertension was 70.5% but was low for dyslipidemia. This could be due to dependency on a lab test to diagnose dyslipidemia versus blood pressure for hypertension, which is easily available at the point of care. However, the diagnosis rate was found to be low (9.5%) for even hypertension. Treatment rate (dyslipidemia 22.1%; hypertension 14.6%) and overall control rate (dyslipidemia 40.1%; hypertension 31%) were poor for both the conditions.

The published data reviewed did not provide information on medication adherence rates in Vietnam, but current practice would suggest that only about 1 in 3 patients are adhering to treatment, whether for hypertension or dyslipidemia. Patients frequently discontinue taking medicine and adopt an unhealthy lifestyle when blood pressure or lipids are controlled. Similar findings were reported by a longitudinal database study from other parts of the world, which reported that around half the patients stop taking medications within the 1^st^ year ^[18]^. Poor treatment adherence compromises the treatment outcome ^[19]^, whether it is pharmacological or lifestyle intervention. The primary reasons for non-adherence to medical treatment in more than 60% of cardiovascular patients include socio-economic factors, inadequate patient education leading to reduced awareness, and lack of motivation, particularly for lifelong medication ^[20]^. Other factors associated with cardiovascular risk reported in the studies were abdominal obesity, diabetes, smoking, poverty, use of alcohol, lead exposure, tobacco use, physical inactivity, and a high-salt and high-fat diet ^[10,11]^. Taken together, all these further predispose patients to develop cardiovascular events and complications. Major contributors to low adherence were identified as lack of awareness and limitations of the healthcare system as described below.

### 4.1. Low health literacy and awareness in Vietnam

Health literacy level in the elderly population, who suffer from a higher prevalence of dyslipidemia and hypertension, has been reported to be low (33%) in Vietnam, which adversely impacts screening, diagnosis, and treatment adherence for the 2 conditions ^[21]^. This was attributed to the low education levels and social-economic status, and hence misconceptions and lack of understanding of disease and treatment in patients and caregivers of aged patients. This is an alarming situation as uncontrolled dyslipidemia and hypertension may lead to complications and adverse cardiovascular outcomes, while the patient remains unaware of the complexity of the situation due to minimal symptoms.

There are limited initiatives to raise awareness among the population regarding hypertension and dyslipidemia. Despite some key initiatives like “The 1^st^ Day project” on hypertension and diabetes treatment with MOH, and “communities for healthy hearts” program by ho chi Minh city provincial health department and the international global health organization program for appropriate technology in health ^[22]^ that aim to improve patient awareness for these conditions, the non-communicable diseases burden is huge, and the risk mitigation is delayed. This could be due to lack of coordination, the expanse of the program, or lack of awareness amongst the user groups. Government initiatives and national programs, such as Vietnam’s National Hypertension Program, focus on screening and treatment rather than awareness and adherence to the medication ^[8]^. More collaborative efforts from different sectors to foster multi-stakeholder involvement and public-private partnership are hence required.

### 4.2. Limitations of the healthcare system and unaffordable treatment for both the conditions

The poor accessibility of medical facilities combined with the restricted availability of adequate treatment has been a hurdle in the management of hypertension ^[7]^. Although the Vietnamese healthcare system has made notable efforts to address this need, it is important to focus on the provision of regular and systematic care that is integrated from the top to the grassroots level ^[8]^. The government finance bears healthcare expenditure for specific population groups ^[8]^; however, the current healthcare system seems to be overwhelmed with the number of patients needing chronic care for dyslipidemia and hypertension and lacks adequate manpower to cater to a large population ^[23]^. Despite public hospitals providing free medicines to the patients, the out-of-pocket expenditure incurred for traveling to the center every month poses a barrier to adherence ^[8]^. Moreover, the family doctors’ network is limited to self-pay service, which is not affordable for all. Furthermore, due to low economic status with poor GDP, limiting purchasing power of Vietnamese patients and lack of affordability for effective treatments adds to the poor adherence. This calls out for subsidized costs of effective medicines in Vietnam, so that costs do not decide the treatment adherence.

### 4.3. Role of PCPs in ensuring adherence to the treatment of hypertension and dyslipidemia

PCPs play a vital role in ensuring the prevention and control of non-communicable diseases ^[8]^. However, the ratio of PCPs to the needy is low, especially in remote areas. High patient load also compromises the consultation time per patient in the outpatient clinics, and the patients are often not educated and counseled about adherence ^[23]^. The paucity of healthcare providers necessitates task shifting from doctors to the paramedical staff. Currently, there is limited opportunity for paramedical staff to provide counseling and patient education because their current role in the healthcare community is primarily to sell or dispense medications.

This situation gets further complicated as PCPs might not be aware of the newer screening and treatment protocols, including emerging guidelines on screening and management of hypertension and dyslipidemia Additionally, cardiovascular risk calculators are not used routinely in practice for evaluating the risk of CVD for the patients in Vietnam ^[24]^. Hence, such a tool is not broadly applied, and PCPs lose the opportunity to accurately assess the risk and initiate medication early enough to prevent the development of cardiovascular complications and events.

### 4.4. Limitations

Limited data were available to synthesize evidence despite a comprehensive search. Expert opinions were taken to supplement the deficits in data for adherence.

### 4.5. Translational value of the review

The local hospitals and primary healthcare clinics play a key role in controlling and managing non-communicable diseases. Therefore, the MoH and medical universities conduct continuing medical education sessions for the PCPs deployed in the district levels hospital and health care professionals in commune health stations. Though the program aims to cover a major proportion the number of healthcare providers trained is still not enough to support a large population. This needs to be taken up more aggressively, which provides larger returns concerning the healthcare expenditure saved, and the control of hypertension and dyslipidemia achieved. Medical universities should also run programs to educate practicing family doctors on newer diagnostic and treatment options.

Adherence is the key to an optimal treatment outcome, and various self-reminding tools for the patients can enhance it significantly. Healthcare professionals-led patient clubs aimed at improving treatment adherence can be established to provide ongoing support to the patients. Providing healthcare finance through Government Medical Insurance for good medications and good medical services for low-income people can remove the financial barrier to accessing appropriate care. It is recommended to channel the health information via official and authorized sources, which is trustworthy and reliable. Here, patients can also directly interact with doctors or healthcare providers. Proper positioning of messages for raising awareness of the population using social networking and media as a medium for educational campaigns can be helpful.

Overall, this study indicates that there is none to scanty data available on treatment in Vietnam. Therefore, strategies and mechanisms to highlight the gaps in the patient journey and identify interventions to bridge the gaps and achieve control of these indications are highly warranted. The review did reflect the data on current country-specific issues prevailing in Vietnam and contributing to the low proportion of patients in various stages of their journey for hypertension and dyslipidemia, which can be useful for designing locally effective and engaging interventions. Policymakers and health system managers can utilize this data to design-focused programs for enhancing awareness in the community and try innovative solutions for increasing adherence, along with updating the knowledge and skills of PCPs.

## 5. Conclusion

Dyslipidemia and hypertension are underlying causes of major cardiovascular events and lifelong morbidity. Low adherence to treatment reduces control of these conditions and puts the patients at enhanced risk of these eventualities. Lack of manpower to cater to large population, socio-economic factors, low awareness, lack of motivation particularly for lifelong medication, and lack of sensitization of primary care physicians regarding updated guidelines were the major factors revealed by the review, which hinder the adherence to the treatment. Focused efforts are needed to prevent the progression of these conditions by using innovative solutions. The data generated in the review will help in reflecting upon issues and barriers to patient care and describing the limitations of primary care in catering to the population suffering from these 2 conditions. This will eventually help in making effective interventions in this regard.

## Acknowledgments

The support provided by the independent reviewer, Utsav Samel from Viatris, is deeply acknowledged. Pfizer Upjohn has combined with Mylan to form Viatris now.

## Author contributions

**Conceptualization:** Pham Manh Hung, Vu Huy Thanh, Hoang Van Syc, Dang Quy Duc, Vuong Anh Tuan, Anh T Q T Tran, Grace E Brizuela, Hieu B Tran.

**Data curation:** Pham Manh Hung, Vu Huy Thanh, Hoang Van Syc, Dang Quy Duc, Vuong Anh Tuan, Anh T Q T Tran, Grace E Brizuela, Hieu B Tran.

**Formal analysis:** Pham Manh Hung, Vu Huy Thanh, Hoang Van Syc, Dang Quy Duc, Vuong Anh Tuan, Anh T Q T Tran, Grace E Brizuela, Hieu B Tran.

**Methodology:** Pham Manh Hung.

**Writing – original draft:** Dang Quy Duc, Vuong Anh Tuan, Anh T Q T Tran, Grace E Brizuela, Hieu B Tran.

**Writing – review & editing:** Pham Manh Hung, Dang Quy Duc, Vuong Anh Tuan, Anh T Q T Tran, Grace E Brizuela, Hieu B Tran.

## Supplementary Material
