# Supplementary material for: Adherence to hypertension and dyslipidemia treatment and its implication on control of cardiovascular disease in Vietnam: A semi-systematic review
Source: Medicine (Baltimore). 2022 Dec 23;101(51):e32137. doi: 10.1097/MD.0000000000032137 (PMC9794305; doi:10.1097/MD.0000000000032137)
Supplement: Supplementary file 1 [file medi-101-e32137-s001.pdf]

Table S1. Definitions of various terms used in the review

A. Hypertension

Awareness	Self-reported or any prior diagnosis of hypertension by a healthcare professional
Screening	Proportion of respondents who had their blood pressure (BP) measured by a doctor or any other health worker
Diagnosis	Patients diagnosed with hypertension by a healthcare professional
Treatment	Use of a hypertension medication for management of the respondent's high BP
Adherence	Proportion of respondents indicating adherence and/or compliance to the prescribed BP medications
Control	Proportion of patients achieving a target BP of ≤140/90 mmHg with treatment
Hypertension	Hypertension was defined as % of respondents having average systolic blood pressure (SBP) ≥ 140 mmHg and/or average diastolic blood pressure (DBP) ≥ 90 mmHg

B. Dyslipidemia

Awareness	Self-reported or any prior diagnosis of high total serum cholesterol by a healthcare professional
Screening	Proportion of respondents who had their cholesterol levels measured by a doctor or any other health worker
Diagnosis	Patients diagnosed with hypercholesterolemia disorder by a healthcare professional
Treatment	Use of medications for management of the respondent's high cholesterol
Adherence	Proportion of respondents indicating adherence and/or compliance to the prescribed cholesterol lowering medications
Control	Proportion of patients achieving a target cholesterol of ≤5.0 mmol/L OR ≤200 mg/dL with treatment
Hypercholesterolemia	Hypercholesterolemia was defined as total cholesterol (TC) of ≥5.0 mmol/L OR ≥200.0 mg/dL
